# Medawar’s Paradox and Immune Mechanisms of Fetomaternal Tolerance

**DOI:** 10.21926/obm.transplant.2001104

**Published:** 2020-03-10

**Authors:** Victoria Rendell, Natalie M. Bath, Todd V. Brennan

**Affiliations:** 1. Department of Surgery, University of Wisconsin School of Medicine and Public Health, Madison, WI, USA;; 2. Department of Surgery, Cedars-Sinai Medical Center, Los Angeles, CA, USA;

**Keywords:** Medawar’s paradox, immune tolerance, reproductive immunology, fetomaternal tolerance

## Abstract

Brazilian-born British biologist Dr. Peter Medawar played an integral role in developing the concepts of immunologic rejection and tolerance, which led to him receiving the Nobel Prize “for the discovery of acquired immunologic tolerance” and eventually made organ transplantation a reality. However, at the time of his early work in tolerance, a paradox to his theories was brought to his attention; how was pregnancy possible? Pregnancy resembles organ transplantation in that the fetus, possessing paternal antigens, is a semi-allogeneic graft that can survive without immunosuppression for 9 months. To answer this question, Medawar proposed three hypotheses of how a mother supports her fetus *in utero*, now known as “Medawar’s Paradox.” The mechanisms that govern fetomaternal tolerance are still incompletely understood but may provide critical insight into how to achieve immune tolerance in organ transplantation. Here, we review current understanding of the immune factors responsible for fetomaternal tolerance during pregnancy and discuss the potential implications for advances in transplantation science.

## Graphical Abstract

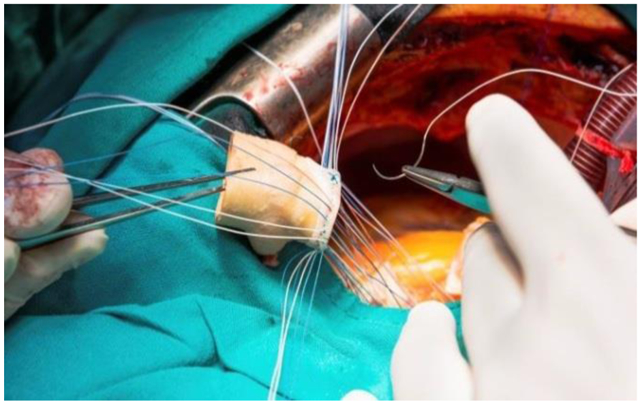

## 1. Introduction

**Figure 1:**
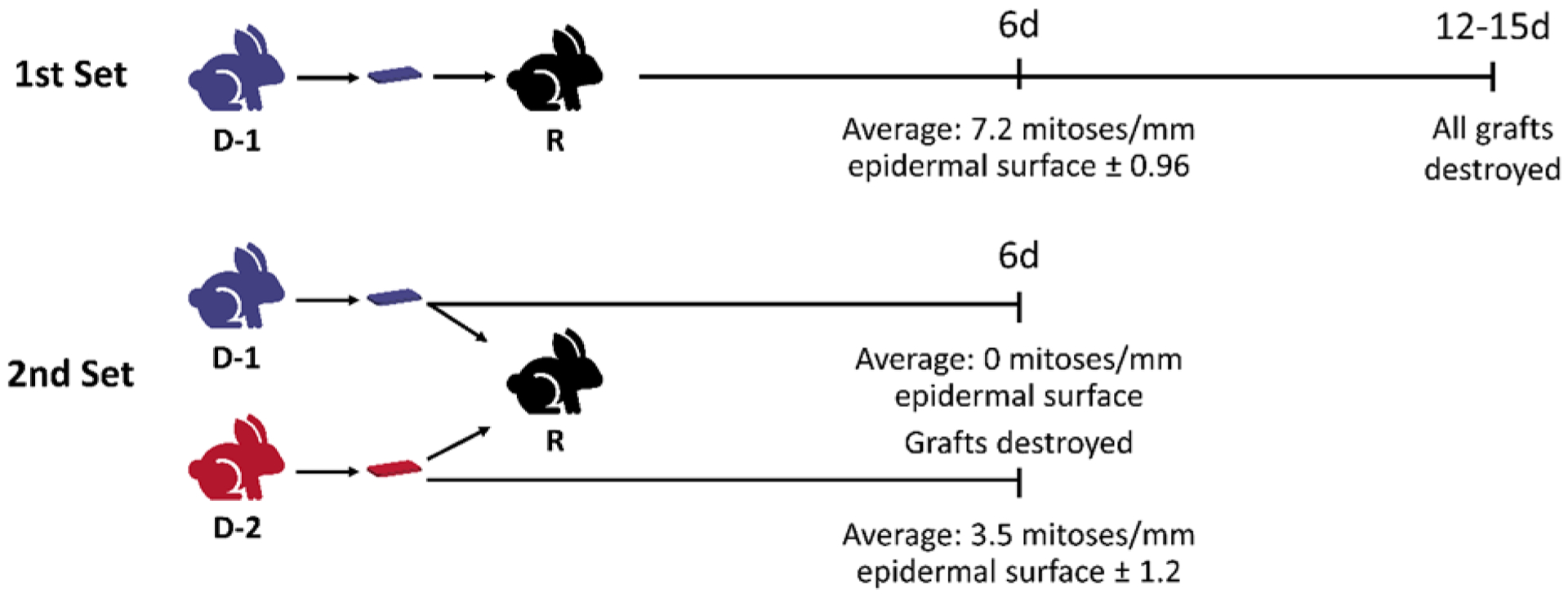
Medawar’s model of allogeneic differences leading to skin graft rejection in rabbits. Medawar grafted skin from one rabbit (rabbit D-1) to another (rabbit R). All grafts were destroyed by days 12–15. Following this, skin grafting was performed from the D-1 rabbit as well as a different rabbit (rabbit D-2) to rabbit R. Cell division was inhibited and graft loss was observed at day 6 for this second grafting that was not observed with the D-2 to R graft. Through these experiments and others, Medawar established that the intensity of homograft rejection was mediated by 1) graft dosage (i.e. amount of skin grafted), 2) previous exposure to grafts from the same donor, and 3) genetic diversity of the rabbits.

Brazilian-born British biologist Dr. Peter Medawar began his exploration of immunology somewhat by chance. He initially studied nerve regeneration; however, his focus changed when he was enlisted after the start of World War II to study why skin grafts between different individuals were rapidly rejected [1, 2]. He used rabbit models of skin transplantation to develop the concept of the immunologic rejection of skin grafts. After demonstrating that skin autografts were successful, he performed a number of experiments with skin grafts clearly demonstrating that all allografts were rejected after a latent period of several days (Figure 1). The speed of graft rejection increased with larger amounts of skin grafted and when the recipient was previously exposure to a skin graft from the same rabbit. Additionally, he noted that variation in graft rejection between rabbit pairs was attributed to genetic differences.

With this change in research focus, he directed his time and energy to investigating immune tolerance and organ transplantation. He was asked to help differentiate monozygotic and dizygotic cattle twins using his skin grafting techniques, assuming that the similar genetic makeup of monozygotic twins would result in successful grafting as occurs with autografts while skin grafting between dizygotic twins would fail due to the genetic diversity essentially making this a homograft [3, 4]. However, in practice this was unsuccessful. Instead, they demonstrated that dizygotic twins (certain to be so because they were different sexes) to a high degree accepted skin grafts from the other, reflecting a type of “desensitization” had occurred. Medawar turned to earlier observations by Ray Owen to explain this phenomenon: that the majority of dizygotic bovine twins have identical red cell antigens [5, 6]. This reflected that a stem cell chimerism in many dizygotic twins exists starting in utero that allows tolerance of skin grafts between the twins later in life [4]. The consideration that stem cells present in embryos could facilitate long-term tolerance led to Medawar’s landmark series of experiments on tolerance in mice initially reported in 1953 and further detailed in his 1956 paper [7–9]. While skin graft survival from donor mice of strain A to CBA strain recipients had an average survival of 11 days, Medawar showed that injecting the fetuses of the mice, or newborn mice during a narrow window after birth, with adult tissue cells from strain A resulted in prolonged and sometimes permanent survival of future strain A skin grafts. Importantly, he also demonstrated that the tolerance was specific to strain A. The results were the same with cellular suspensions from a variety of adult tissues, including kidney, liver, testis and spleen. He also showed this tolerance phenomenon held for skin grafts in other animals (e.g. chickens, ducks, rats) and that injecting whole blood or concentrated leukocytes was also effective for tolerance induction.

## 2. Medawar’s Paradox and Human Pregnancy

Medewar’s work and collaborations eventually led to the ability to successfully transplant human organs. However, at the time of his early work in tolerance, a paradox to his theories was brought to his attention; how was pregnancy possible? Although organ transplantation and pregnancy are clearly different processes, pregnancy resembles organ transplantation in that the fetus, possessing paternal antigens, is a semi-allogeneic graft and immunologically foreign to the mother. For example, mothers cannot accept transplants from their children without immunosuppression. So how do they tolerate the fetus prior to birth? As Medawar stated, “The immunological problem of pregnancy may be formulated thus: how does the pregnant mother contrive to nourish within itself, for many weeks or months, a foetus that is an antigenically foreign body?”

To answer this paradox, Medawar proposed three hypotheses of how a mother supports her fetus *in utero*, (1) *anatomical separation* between mother and fetus by the placenta, (2) *immaturity of fetal antigens*, impairing their ability to elicit a maternal immune response, and (3) *immunological inertness of the maternal immune* system during pregnancy [7, 10]. The mystery of successful gestation is often referred to as “Medawar’s Paradox”.

In 1960 Medawar shared the Nobel Prize for Physiology or Medicine with Sir Macfarlane Burnet “for discovery of acquired immunological tolerance.” However, while Medawar’s original hypotheses have been proven incorrect, modern-day investigations have not completely resolved the paradox of fetomaternal immune tolerance. In essence, pregnancy involves a semi-allogeneic transplant that survives without immunosuppression for 9 months. The mechanisms that govern fetomaternal tolerance are still incompletely understood but may provide critical insight into how to achieve immune tolerance in organ transplantation. Here, we review current understanding of the immune factors responsible for fetomaternal tolerance during human pregnancy using Medawar’s original hypotheses as a framework for discussion, and we discuss the potential implications for advances in transplantation science.

### 2.1 Anatomical Separation

Although the placenta does present a physical barrier between mother and fetus as Medawar initially proposed, it is not an impermeable barrier. Rather, fetal cells and DNA are detected in the peripheral maternal circulation, and non-inherited maternal antigens (NIMAs) are present on cells detected in a child years after birth emphasizing there is an exchange that occurs across the interface. Additionally, allospecific maternal T cells proliferate during pregnancy, further indicating maternal recognition of fetal antigens does occur.

#### 2.1.1 Human Placental Anatomy

**Figure 2:**
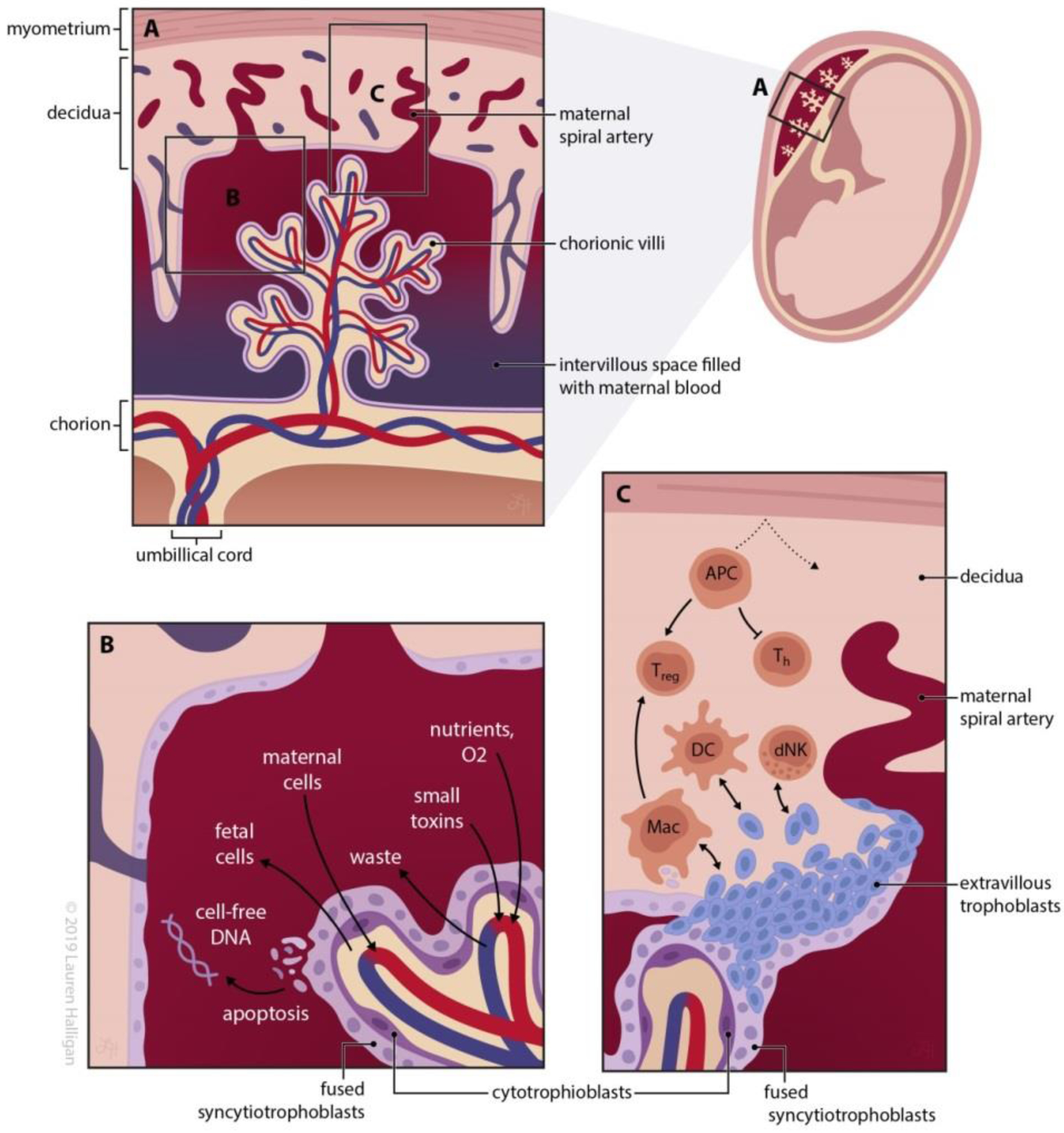
The fetomaternal interface of the placenta is the primary site of maternal and fetal immune interaction. A) As a part of implantation, the fetal trophoblasts of the human placenta invade the maternal decidua to form chorionic villi, which are in direct contact with maternal blood and permit nutrient and oxygen exchange to nourish the growing fetus. Cell turnover is a hallmark of the chorionic villi as the undifferentiated cytotrophoblasts replenish the external fused syncytiotrophoblast layer and differentiate into extravillous trophoblasts, which are known to play key roles in driving maternal spiral artery remodeling and communicating with maternal cells throughout the pregnancy. B) The placenta acts as a semi-permeable membrane, fulfilling its primary function of extracting nutrients and oxygen from the maternal blood supply and permitting the return of waste. Apoptosis of the syncyctiotrophoblasts results in apoptotic bodies containing fetal genetic material to be returned to the maternal circulation as “cell-free DNA”. There is also a small amount of transfer of fetal cells to the maternal circulation as well as maternal cells to the fetus, known as microchimerism. C) Key crosstalk between fetus and mother involves several immune cells. Maternal APCs are trapped in decidua due to lack of lymphatic vessels, which prevents activation of maternal T cells and destruction of the fetus. Decidual macrophages and decidual NK cells are key regulatory cells at the interface. CD4+ T cell proliferation is downregulated and activated CD8+ T cells undergo apoptosis due to the presence of soluble non-classical MHC class I HLA-G, and regulatory T cells (T_regs_) play a key role in establishing feto-maternal tolerance at the placental interface as well.

In order to better understand the interaction between fetal and maternal immune systems, it is important to understand placental anatomy and function. During pregnancy the human placenta not only functions as the lungs, gastrointestinal system, kidneys and liver of the fetus, but it also undergoes continuous modifications as its purpose changes throughout trimesters [11, 12]. After fertilization, the uterine epithelium prepares to accept the implantation of the blastocyst, transforming into the specialized decidua as the blastocyst invades. The decidua is characterized by its decidual stromal cells, glandular epithelial cells, endothelial cells and maternal leukocytes. The blastocyst’s trophoectoderm cells become the placenta, which develops during the early first trimester. Fetal trophoblasts are the primary tissue in contact with the maternal decidua and therefore the maternal immune system (Figure 2A) [12, 13]. Chorionic villi form the outer edge of the placenta and are outpouchings of stroma and blood vessels surrounded by a layer of cytotrophoblasts, the undifferentiated progenitor trophoblasts, and an outer layer of syncytiotrophoblasts. The syncytiotrophoblast are fused multinucleated cells that form a barrier against the maternal decidual cells, participate in nutrient and oxygen transport from the surrounding maternal blood, and secrete hormones.

In addition to replenishing the syncytiotrophoblast layer, cytotrophoblasts also differentiate into cells termed extravillous trophoblasts (EVTs), which concentrate at the end of each chorionic villus and enter the maternal decidua and interact with maternal vascular and decidual cells, resulting in a remodeling of uterine spiral arteries that allows blood flow to the fetus for ongoing support and development [14, 15]. EVTs play an important role in communication with maternal cells as the dual placental function of immune protection and provisions of nutrients to the fetus continues throughout the duration of the pregnancy. At the 10^th^–12^th^ week of pregnancy, the intervillous space around the chorionic villi becomes bathed in maternal blood, allowing continued nutrient supply to the developing fetus (Figure 2).

This interface between the placenta and the decidua is certainly not an impermeable barrier. By design, nutrients and oxygen cross the placenta from the decidua. Additionally, various chemical toxins are able to transfer across the placenta, particularly when their molecular weight is <1 kDa, which is particularly relevant when considering pharmaceutical therapy for a pregnant woman or the susceptibility of the fetus to the exposure of the women to environmental or other toxins [16]. Apoptosis of syncyctiotrophoblasts in the chorionic villi causes the release of cellular components into the maternal circulation [17]. These apoptotic bodies contain genetic material from the placenta that the majority of the time mirrors the fetal genotype, which has now been isolated from maternal blood samples and termed “cell-free DNA.” Analysis of this DNA has provided important information about the health of the fetus and the placenta. While very selective, the bi-directional exchange of cells and cellular materials across the fetomaternal interface has been demonstrated (Figure 2B). It is important to remember the interface of the placenta with the maternal decidua is dynamic throughout the 9 months of gestation.

#### 2.1.2 An Imperfect Barrier: Microchimerism

Microchimerism occurs when fetal cells are found in maternal tissues and circulation, and/or maternal cells are present in their offspring.[18]. In maternal-fetal cellular trafficking, the trophoblast allows bidirectional movement of stem cells and leukocytes. Fetal cells that have been detected in maternal circulation also include trophoblasts, granulocytes, lymphocytes, and nucleated red blood cells [19]. These cells and fetal DNA have been found in peripheral circulation and in maternal tissues decades following pregnancy. Additionally, maternal cells can be found in immunocompetent adult offspring [20–23].

Long-term microchimerism has several immunological implications serving both pathologic and protective functions. In parous women, long-term microchimerism has been associated with autoimmune disorders but has also been found to offer protection against certain cancers [24, 25]. Pediatric immune-mediated diseases such as neonatal lupus syndrome has been associated with microchimerism in immunocompetent offspring [23]. More importantly, maternal microchimerism has been identified as a mechanism for maternal tolerance towards fetal inherited paternal antigens (IPAs) whereas fetal microchimerism promotes tolerance towards NIMAs in the offspring [26, 27].The fetal tolerance towards NIMAs may be lifelong; however, maternal tolerance towards IPAs may be short-lived [28]. This tolerance has broader implications outside of pregnancy as seen in hematopoietic stem cell (HSC) transplantation, in which Ichinohe et al. demonstrated a lower incidence of graft-versus-host disease with HLA-haploidentical HSC transplant from a microchimeric IPA/NIMA-mismatched donor [26]. The mechanism behind this tolerance is thought to be related to a deletion of IPA/NIMA reactive T cells and an upregulation of T_regs_ [26].

As described above, the bi-directional nature of the placenta facilitates tolerance between mother and fetus. Similarly, violation of this barrier that occurs in pre-term labor or trauma results in a breakdown of fetomaternal tolerance. Pre-term labor frequently occurs after fetal surgery, which is performed with the goal of improving morbidity and mortality for severe or fatal congenital anomalies. Normally, maternal fetal cellular trafficking results in fetal cells in maternal circulation and maternal cells in the fetal circulation. Maternal cells in fetal circulation leads to generation of fetal T_regs_, which then suppress fetal effector T cells from binding to maternal antigens [27, 29, 30]. However, murine models have indicated that when the placental membrane is interrupted during fetal intervention, trafficking of maternal T cells into the fetus increases [31]. When allogeneic fetuses were injected with saline in-utero, an increase in maternal T cell migration to the myometrium and an increase in effector cytokines was noted. Lastly, murine fetuses were injected with foreign paternal allele and a specific loss in pups that were genetically different from the mother was seen whereas genetically identical pups were preserved. These findings demonstrate the significant change that occurs in fetomaternal tolerance when the placental membrane is disrupted in murine models [31]. These results seen in animal models cannot be directly extrapolated to human pregnancy; however the concept of changes in fetomaternal tolerance as a result of placental disruption is important to note.

### 2.2 Immaturity of Fetal Antigens

Medawar’s second hypothesis behind fetomaternal tolerance focuses on the immaturity of fetal antigens, which subsequently prevents the maternal immune system from mounting an attack against the fetus. EVTs do display unique major histocompatibility complex (MHC) antigens, including non-classical human leukocyte antigen-G (HLA-G) and are deficient in MHC class II antigens. However, the fetal immune system does not consist entirely of immature antigens, but rather is composed of antigens not recognized by the mother, which results in downstream effects on both innate and adaptive immune responses. Here we discuss fetal antigens and the immune changes that occur in the fetomaternal environment.

The fetal trophoblast is an immunologic barrier that promotes immuno-tolerance between mother and fetus through several mechanisms including absence of classical HLA class I and II expression in the fetus, altered natural killer (NK) cell and T cell populations and functions, low tryptophan levels, and high progesterone levels [32–35]. A summary of crucial first trimester maternal immune cell populations and cytokines are presented in Table 1.

#### 2.2.1 Altered MHC Expression And Fetal Immunogenicity

Human EVTs express the classical MHC class I antigen HLA-C, the non-classical class I antigens HLA-E, -F, and –G, and do not express MHC class II antigens (HLA-DP, -DQ, -DR) (Table 1). Due to lack of expression of classical antigens in the fetus, the maternal immune system cannot mount a response to paternal HLA present in the fetus, thereby preventing direct alloantigen recognition [38–41]. However, as described above, maternal antigen presenting cells (APCs) could potentially process and present conceptus-derived antigens on maternal MHC molecules, which would then activate maternal T cells (indirect alloantigen recognition) [42]. This indirect antigen presentation pathway does not occur, however, due to the fact that maternal APCs remain trapped in the decidua. Maternal APCs may be trapped due to lack of lymphatic vessels in the uterus or potentially due to changes in the decidual extracellular matrix, which then prevents maternal APCs from migrating to lymph nodes as seen in murine models [43]. Further studies are needed to determine if this phenomenon also occurs in humans. Additionally, human decidua has been found to have fewer DCs compared to human endometrium [44]. This paucity of maternal APCs and overall lack of functionality represents one possible mechanism for fetomaternal tolerance [43]. The altered fetal MHC antigen profile at the fetomaternal interface has several downstream effects as well on both innate and adaptive immune responses in this environment. MHC class I antigens HLA-A and HLA-B play a role in antigen presentation to T cells and NK cells. EVTs express HLA-C, HLA-E, and HLA-G, but lack HLA-A and HLA-B. HLA-C expression at this interface prevents maternal NK cells from attacking the fetus [15, 45, 46]. At the same time, the presence of the non-classical MHC class I antigen HLA-G in soluble form has immunosuppressive effects through the downregulation of CD4+ T cell proliferation and the induction of activated CD8+ T cell to undergo apoptosis [47, 48]. Additionally, HLA-G expressed on cell surfaces binds to inhibitory cell surface receptors resulting decidual APCs and decidual natural killer cell (dNK) inhibition, which further prevents the attack of the maternal immune system against the fetus [12, 41, 49]. The lack of MHC class II antigens is also a key characteristic of the fetomaternal interface. MHC class II antigens are recognized by CD4+ helper T (T_H_) cells, which in turn participate in antibody and cytotoxic T cell immune responses. By not having MHC class II expression in the fetus, the maternal immune system is not able to initiate the T_H_ cell-mediated immune response that would occur from paternal foreign antigen recognition (Figure 2) [12]. Furthermore, the lack of functional maternal APCs at the feto-maternal interface is critical since APCs do express MHC class II and therefore would normally stimulate T_H_ cells. As a result, violations of the fetomaternal interface could result in an escape of maternal APCs into the fetus and ultimately inciting an immune reaction [31, 43].

#### 2.2.2 Inflammatory Mediators at the Fetomaternal Interface Preventing Fetal Destruction

Although fetal cells express some level of antigen immaturity displayed by altered MHC expression, fetal cells also actively play a role in cross-talk with maternal cells, which promotes a tolerogenic environment locally. At a cellular level, T cell dependent inflammatory responses are suppressed due to decreased levels of tryptophan seen in pregnancy. Tryptophan is degraded in syncytiotrophoblasts, invasive EVT and macrophages by the enzyme Indoleamine 2,3-dioxygenase (IDO). When IDO degrades tryptophan, cells are able to suppress T cell activity *in vitro* [50]. Conversely, when an IDO inhibitor is given, this suppressive effect is reversed. Similarly, when treatment with an IDO inhibitor is given, syngeneic fetuses are not rejected [51]. Therefore, it is thought that tryptophan metabolism in trophoblasts and APCs in the placenta protect the fetus by inhibiting T cell activation. Fetal EVT also contain a high concentration of IDO during the first trimester and at term, which are the fetal cells located closest to the maternal immune system. The close proximity of EVT to the maternal immune system demonstrates the critical role IDO likely plays in down-regulating maternal T cell responses [52]. Additional mechanisms of protection through tryptophan metabolism have been proposed including T cells apoptosis due to release of toxic tryptophan breakdown products such as kynurerine, 3-hydroxykynurenine, and 3-hydroxyanthranilic acid [50, 53]. Although macrophages primarily produce IDO, monocyte-derived DCs are also found to produce IDO. DCs are potent activators of T cells; however, DCs are also important regulators of the immune system and IDO production represents a mechanism for how DCs inhibit cellular immune responses [54].

The importance of IDO and tryptophan metabolism has been demonstrated in murine allogeneic pregnancy models. Pregnant mice carrying allogeneic or syngeneic concepti were treated with IDO inhibitor, which resulted in a significant loss of concepti in the allogeneic but not syngeneic pregnancies. To determine the importance of maternal lymphocytes in rejection, *RAG-1*^−/−^ mice, which prevents the development of lymphocytes, with allogeneic or syngeneic pregnancies were again given an IDO inhibitor. Both syngeneic and allogeneic pregnant females delivered healthy litters. However, when given a reconstituted T cell population, allogeneic, but not syngeneic, pregnant mice lost all their concepti. This study by Munn et al. demonstrates that not only is tryptophan metabolism crucial in the inhibition of T cells, but when not inhibited, maternal T cells are responsible for rejection. Moreover, this experiment indicates that the fetomaternal interface is not an anatomical barrier and is not antigenically immature, but rather survival of allogeneic fetuses results from immunomodulation at this interface [55]. The IDO inhibitor used was Munn et al. has been found to have an inhibitory effect on human placental IDO, suggesting that this murine model may also be applicable to human pregnancy [56]. This enzyme has been localized to the cytoplasm of syncytiotrophoblasts (and not expressed at the brush border membrane) indicating that tryptophan must enter the cell in order to be metabolized. Tryptophan metabolism may therefore be regulated by transmembrane transport, which in turn may represent the mechanism for maternal immune suppression to the fetus [55, 56]. Other studies have demonstrated normal-sized litters in matings involving IDO-deficient males and females. This finding suggests that although IDO may play a role in maintaining allogeneic pregnancies, other compensatory immunosuppressive mechanisms likely exist to prevent rejection [57].

#### 2.2.3 Promoters of Apoptosis

paraAs seen in the tryptophan degradation pathway, apoptosis is an important mechanism utilized by cells in the placenta to facilitate fetomaternal tolerance during implantation and throughout pregnancy [58]. Fas ligand (FasL) and TNF-related apoptosis-inducing ligand (TRAIL) are both members of the TNF superfamily and play crucial roles in apoptotic cell death [59, 60]. FasL is expressed not only on activated immune cells, but is also expressed on the surface of other cells in immune privileged sites including the eye, brain, testis, and placenta [61]. TRAIL has previously been established as playing a role in apoptotic cell death in tumor cells, but has recently been found to be involved in immune surveillance, intra-thymic negative selection, and suppression of autoimmunity [62]. Together, FasL and TRAIL are intracellularly expressed in syncytiotrophoblasts and secreted as part of placental exosomes. Once secreted, these death messengers form a complex that are then able to induce apoptosis in activated lymphocytes that could pose a threat to the fetus [63, 64].

### 2.3 Immunological Inertness of the Maternal Immune System

Medawar initially hypothesized that maternal immune system inertness during pregnancy would help explain tolerance of the fetus. While pregnant women do have increased susceptibility to infection and demonstrate alterations in their immune profiles, these modifications are nuanced and do not lead to a state of immunological inertness demonstrated by the ability of pregnant women to still mount an immune response and respond robustly to various pathogens [65]. During pregnancy, maternal immune cell populations and functions are altered both locally—at the fetomaternal interface—and systemically. In this section, we review these alterations and how they contribute to tolerance of the fetus.

#### 2.3.1 Maternal Immune Cells at the Fetomaternal Interface

The presence of maternal immune cells at the fetomaternal interface is important for establishing a functional exchange, and the cross-talk between trophoblasts and maternal immune cells helps direct normal placentation. A variety of maternal immune cells are present at the interface and participate in this role; however, the characteristics of the cell population are unique in this environment (Figure 2C).

Although the changes in immune cell populations and MHC expression differences seen on fetal cells help to shed light on why a fetus is not “rejected,” this phenomenon is still not completely understood. Immature MHC and the lack of classical MHC expression (except for HLA-C) does not exclude rejection. This finding is similar to what is seen in an HLA identical transplant, in which rejection may still occur due to minor (non-MHC) antigens contributing to rejection [66].

##### The Role of Adaptive Immune Cells at the Fetomaternal Interface.

The principal driving force of why mother and fetus are able to co-exist is the establishment of immuno-tolerance [32]. Multiple adaptive immune cell types play a role in establishing this balance, but key cell mediators are T_regs_ and T_H_ cells. In addition to differences in antigens present at the fetomaternal interface, it is also important to note the composition of immune cells and their unique functions at this junction.

CD4+CD25+FoxP3+ T_regs_ play a significant role in facilitating fetomaternal immuno-tolerance, which begins before pregnancy during the menstrual cycle. Female T_reg_ population increases just prior to ovulation, a time when the female could potentially be exposed to foreign paternal-fetal antigens [67, 68]. As seen in murine models, this T_reg_ expansion correlates with a peak in serum estradiol levels; additionally, progesterone levels continue to rise after ovulation and together with cytokines IL-2 and TGF-b, have increased ability to induce Foxp3 expression, and therefore, increase the T_reg_ population [69]. Due to the fact that T_regs_ are present in the uterus in increased numbers even prior to implantation, their expansion is likely stimulated by estrogens and trophoblastic cytokines [70]. Correspondingly, women who experience recurrent miscarriages have been observed to possess a smaller T_reg_ population [71].

In addition to T_regs_, the PD1-PDL1 negative costimulatory pathway plays an important role in developing and maintaining tolerance by regulating the balance between T_regs_ and pathogenic T cells [72]. PDL1 blockade in a murine fetomaternal tolerance model resulted in an inhibition of apoptosis of alloreactive T cells, an increase in T_reg_ apoptosis, and an increase in T_H_17 cells. The loss of regulatory function seen with PDL1 blockade has been shown to result in expansion of effector cells T_H_1 and T_H_17, which leads to fetal rejection and a reduction in litter size [72, 73]. These studies demonstrate that PDL1 inhibition leads to a breakdown of fetomaternal tolerance due to an increase in T_H_17 cells and decrease in T_regs_. This balance between T_H_17 cells and T_regs_ is crucial to maintaining fetomaternal tolerance.

The PD1-PDL1 checkpoint also plays a significant role in the ability of cancer cells to evade immune system detection. Human trophoblastic cells are similar to malignant cells in that they are both able to invade normal tissue including blood vessels and they are able to avoid destruction by the host immune system. Veras et al. investigated PDL1 expression in order to determine if PDL1 contributed to immune system evasion in pregnancy and in gestational trophoblastic diseases. PDL1 was upregulated in syncytiotrophoblast and in intermediate trophoblastic cells located at the implantation site, which suggests that the PD1-PDL1 checkpoint may be an additional mechanism used to establish fetomaternal tolerance [74].

Fetal-specific CD8+ cytotoxic T cells have been detected in women with a previous pregnancy, and in order to maintain tolerance, it was thought that these cells underwent Fas/FasL-mediated apoptosis during early pregnancy. [75]. However, a human study by Lissauer et al. demonstrated that fetal-specific CD8+ T cells were present in half of all pregnancies with fetal-specific cell populations increasing throughout pregnancy and remaining in the post-natal period. Not only were these effector T cells still present, but were still able to proliferate, secrete Interferon gamma (IFN-γ), and lyse target cells in vitro [76]. This finding suggests that other mechanisms aside from apoptosis must occur in order for fetal cells to avoid being targeted by maternal T cells. Although fetal-specific T cells were functional in vitro, these cells may be functionally attenuated or anergic in vivo. Tregs may suppress the activity of these fetal-specific T cells in vivo. Fetal cells may evade the maternal immune system through HLA down-regulation on trophoblasts and syncytiotrophoblasts. [77]. Additionally, lack of CD8+ T cell infiltration into the decidua has been associated with pre-eclampsia demonstrating that alloreactive T cells confer a survival advantage during pregnancy [78].

##### The Role of the Innate Immune System at Fetomaternal Interface.

In addition to adaptive immune system changes throughout pregnancy, the innate immune system plays a significant role, namely through decidual macrophages and dNK cells. These maternal leukocytes, recruited by decidual stromal cells and trophoblasts, are present in the decidua throughout pregnancy with the highest concentrations seen during the first trimester [79–81]. dNK cells comprise 70% of the leukocytes present in decidual tissue during the first trimester where they are the critical mediators of trophoblast invasion and the remodeling of spiral arteries during decidualization and implantation. dNK cells are modulated by placental EVT to promote fetomaternal tolerance and to prevent destruction of the fetomaternal inferface. A tolerogenic environment is created through the binding of KIR, CD94/NKG2A, and ILT2 receptors located on dNK cells to HLA-C, -E, and –G, respectively [82]. Placental EVTs express the non-classical MHC molecule HLA-G, which can bind to dNK killer cell immunoglobulin (Ig)-like receptors (KIR) KIR2DL4 and LILRB [12]. These interactions protect trophoblasts from dNK cytotoxicity in the setting of low-level expression of conventional MHC molecules [83–85]. Upon binding to HLA-C, KIR inhibits the cytotoxic activity of dNK cells thereby preventing trophoblastic lysis. HLA-E is located on trophoblasts and maternal cells; therefore, the destruction of these cells is prevented when HLA-E binds to the inhibitory receptor CD94/NKG2A. ILT2 binding results in the secretion of inflammatory and pro-angiogenic factors including IL-1B, IL-2, IL-8, and TNF-a [82, 86].

However, unlike peripheral NK cells which are low producers of cytokines and have potent cytotoxic capabilities, dNK produce high levels of cytokines, growth factors, and angiogenic factors and display low NK cytotoxicity [87]. Cytokines secreted by dNK cells include macrophage inflammatory protein (MIP), granulocyte-macrophage colony-stimulating factor (GM-CSF), vascular endothelial cell-C (VEGF-C), and placental growth factor [88, 89]. These secretions aid in the remodeling of the decidua and spiral arteries, recruit trophoblasts to invade via IL-8, and increase maternal blood at the site of implantation [85, 90, 91]. As evidence of the importance of dNK cells, endometrial biopsies of patients with unexplained infertility display a paucity of dNK cells compared to fertile counterparts [92]. Interestingly, recent data points to a subset of dNK cells expressing high levels of NKG2C and LILRB1 receptors and present in much higher levels in multigravid women compared to primigravid women. This subpopulation of dNKs secretes higher levels of IFN-γ and VEGFα, which enhance performance for angiogenesis and vascular remodeling during placentation in subsequent pregnancies [93].

Additionally, decidual macrophages play an integral role as the primary APC at the fetomaternal interface during early pregnancy. Decidual macrophages act as regulatory cells and have an anti-inflammatory phenotype [82]. They aid in spiral artery and trophoblast remodeling and in angiogenesis via the production of VEGF and matrix metallopeptidase 9 (MMP9). By phagocytosing apoptotic trophoblasts, decidual macrophages prevent the activation of decidual pro-inflammatory pathways. With the production of IDO, decidual macrophages are able to inhibit T cell activation [94, 95].

Together, decidual macrophages and NK cells contribute to maternal tolerance through the modulation of effector T cell populations and the promotion of regulatory phenotypes of leukocytes that are present at the interface. At the beginning of pregnancy, effector T cell populations are small in comparison to decidual macrophages and dNK cells potentially due to silencing of T cell chemokines in stromal cells. Decidual leukocytes, EVT and stromal cells promote the differentiation of monocytes and T cells into M2 macrophages and T_regs_ through the production of granulocyte colony-stimulating factor (G-CSF), IL-10, and TGF-β [96–98].

#### 2.3.2 Systemic Immunomodulatory Changes in the Mother

While we know that a variety of immune factors at the fetomaternal interface permit tolerance to the semi-allogeneic fetus, there is also evidence of systemic immune alterations in the mother during pregnancy. For example, pregnant women with autoimmune diseases tend to have either improvement or worsening of their autoimmune disease during pregnancy depending on the nature of the disease, and pregnant women are more susceptible to morbidity and mortality as a result of infection by certain pathogens, such as influenza, *Listeria monocytogenes*, and *Varicella zoster* [99].

An initial more simplistic theory for systemic changes in immunity of the mother related to a thought that the immune cell distribution and cytokine production shifted the T_H_1/T_H_2 balance towards a more tolerogenic T_H_2 profile [100–103]. Although the T_H_2 bias of the uterine environment has been demonstrated, but the extension of this observation from the fetal maternal interface to the systemic maternal immune system is controversial and has not been supported by the most recent studies of maternal immune profiles during pregnancy [99]. Indeed, throughout pregnancy shifts in maternal immune cell populations are observed, including some inhibition of immune activity but also immune activation [104]. The Viral Immunity in Pregnancy (VIP) Project provided longitudinal assessments of peripheral immune cell populations and cytokine production in women throughout pregnancy with a comparison to two postpartum time points: 5–6 weeks and 6 months postpartum [105, 106]. Through their analysis of maternal peripheral blood, they demonstrated an overall peripheral immune cell population profile that reflects an increase in innate immune cell populations, such as DCs, monocytes, and neutrophils combined with a decrease in T cells, NK cells and B cells overall. They additionally demonstrated alterations in immune cell *function* reflected by levels of cytokine production in response to various stimuli. They demonstrated an overall decrease in CD4+ and CD8+ T cells as well as a decrease in toxic shock syndrome toxin (TSST)-stimulated production of both T_H_1 and T_H_2 cytokines in CD45RA+ (naïve) T cells. Peripheral NK cell populations were highest in the first trimester and decreased after the 20^th^ week of pregnancy, reflecting the early need for uterine NK cells during placentation. Similarly, there was a decrease in CD19+ cells B cells later in pregnancy. In the analysis of cytokine expression throughout pregnancy, they demonstrated variation in cytokine profiles between individual women. However, when comparing the cytokine levels for the same woman at the 6 week and 6 month postpartum time points, they found high concordance of the cytokine profiles, suggesting distinct patterns of baseline serum cytokine expression exist at the individual level. Even with this individual-level variation, they demonstrated clear trends in peripheral cytokine profiles throughout pregnancy compared to postpartum levels. TNF-α and the growth factor G-CSF were all elevated throughout the three trimesters. IFNγ, MCP-1, VEGF, and Eotaxin were all decreased throughout pregnancy with the exception of INFγ, which was only decreased in the 2^nd^ and 3^rd^ trimesters. These findings support a more complex systemic immune response to pregnancy in women than a simple overall shift towards T_H_2 profiles and reflects the need for a pregnant woman to be able to defend against infection. The results of the VIP project suggest that in pregnancy there is a shift towards bolstering the innate immune system while sacrificing some protection to viral infections.

Continued work in this area has provided additional information regarding the complex immune shifts in pregnancy. Aghaeepour et al. recently helped to shed additional light on the temporal variations in systemic immune responses in pregnant women, using a mass cytometry technique to create a high parameter functional profile of the peripheral immune cells and cell signaling throughout normal pregnancy [107]. In this study, 21 pregnant women had serial blood samples obtained (early, mid and late pregnancy) along with a control sample at 6 weeks postpartum. Mass cytometry assays were run to simultaneously interrogate multiple signaling pathways in distinct cell subsets spanning the entire immune system at each time point to understand the systemic “immune clock” of pregnancy. A cell signaling based algorithm Elastic Net (csEN) was used to create a model of the immune features at all time points to predict the timing of immune changes during a pregnancy, and the model was tested on an additional 10 enrolled pregnant women with good performance demonstrated. Through these analyses, Aghaeepour et al. confirmed and further delineated the initial observations of the VIP study related to temporal immune changes in pregnant women with more information about cell signaling pathways in immune cell populations. They also demonstrated increased immune cell populations, such as increased levels of circulating neutrophils in pregnancy with enhanced responses to stimuli. They also found circulating maternal DCs to have a higher expression of tolerogenic surface proteins (such as PD-L1) and decreased TLR4 signaling in response to LPS stimuli. With regards to T cell function, they actually found some endogenous signaling pathways to be increased in response to certain stimuli rather than the overall consistent decrease in function seen in earlier studies. Most intriguing was the progressive increase in STAT5ab signaling in multiple T cell subsets and the observation of an increase in circulating IL-2 levels in pregnancy. It is known that STAT5ab activity dependent on IL-2 is essential for the development of T_regs_, which implicates this pathway in a potential role for increased presence of T_regs_ in pregnancy. Maternal peripheral immune cell population alterations and cytokine production throughout gestation are summarized in Table 2.

The main known drivers of these systemic immune changes in pregnant women include the hormones produced during pregnancy that are known to alter immune response-related transcription signaling at many levels. Estrogen and progesterone receptors are present on a wide variety of cell types, including lymphocytes and APCs. During pregnancy, human chorionic gonadotropin (hCG), first produced by the blastocyst and later by trophoblasts, plays a major role early in pregnancy by promoting implantation and placentation and then stimulating progesterone production [108]. The fetus plays an important role in tolerance through the production of hCG. hCG has been shown to stimulate the production of IL-10^+^ Breg (B10) and IL-35^+^ Breg cells, which are known tolerogenic cells that function to downregulate effector cells.[109] Progesterone and estrogen levels increase through gestation while hCG levels peak around the 11^th^ week of pregnancy.

Estrogen is present in pregnant women in two forms, estradiol (E2) or the classic form of estrogen present in all pre-menopausal women and estriol (E3), which is produced by the fetoplacental unit and represents 90% of all estrogens produced in pregnancy [110]. Estrogens are implicated in multiple immune changes known to occur during pregnancy, particularly the later changes in the third trimester when the estrogen levels are highest. Centrally, estrogen causes thymic involution, leading to decrease in T cell development [111]. Additionally, estrogen suppresses B cell lymphopoiesis [112]. In addition to regulation of immune cell production, estrogen has been shown to drive bone marrow precursor cells to formation of CD11c+ DCs and decrease antiviral responses in addition to augmenting T_H_2 responses including IFNγ production and expand T_reg_ populations [110].

Progesterone is mainly produced by the corpus lutea of the ovaries as well as the placenta during pregnancy and has a largely anti-inflammatory role during pregnancy, inhibiting TLR-induced cytokine production and promoting T_H_2 immune responses while inhibiting T_H_1 immune responses [113–115]. These effects of progesterone on T cells are mediated by inhibition of the NF-κβ pathway as well as progesterone-induced blocking factor (PIBF) [116]. Progesterone has been shown to have varied effects on T_reg_ populations in humans versus mice. Progesterone stimulates the release of CXCL10, a chemokine implicated in the localization of T_H_2 cells to the placenta and is also responsible for upregulation of HLA-G expression [115].

## 3. Implications of Immune Changes of Pregnancy for Future Maternal Pathology

The interactions between maternal and fetal immune systems that occur during pregnancy have significant clinical implications. Autoimmune diseases occur more commonly in women following their reproductive years, which is thought to be due to maternal fetal cell trafficking during pregnancy. Specifically, women with systemic sclerosis have increased rates of fetal microchimerism, and fetal cells are commonly detected in women diagnosed with Hashimoto’s thyroiditis, Graves’ disease, and scleroderma [18, 117–119]. Conversely, maternal cells have been detected in higher frequency in children with neonatal lupus syndrome, type I diabetes, juvenile dermatomyositis, biliary atresia, and Hirschsprung’s disease [18]. However, it has not been determined if the presence of maternal or fetal cells contribute to autoimmunity or to tissue regeneration. Fetal cells found in mothers have stem-cell like properties as they are capable of differentiating into various cell types including blood and skin, and are found to contribute to tissue regeneration in maternal liver and kidneys after injury [120, 121]. Alterations in immune cell populations during pregnancy have been linked to maternal disease, including low postpartum T cell levels in mothers who develop postpartum psychosis and high CD4/CD8 T cell ratios with higher levels of activated T cells in women who develop postpartum thyroiditis [122, 123].

Maternal microchimerism has clinical significance as seen in liver transplantation between mother and child for biliary atresia. As previously mentioned, maternal cells in fetal circulation result in the development of fetal T_regs_, which promote tolerance to NIMAs [30]. Increased maternal microchimerism is seen in livers of neonates affected by biliary atresia [29]. In a retrospective review by Nijagal et al., pediatric patients who received a liver transplant from their mother for biliary atresia had significantly lower rates of graft failure and need for retransplantation compared to those who received liver transplants from their father. This finding indicates that maternal microchimerism likely plays a long-term role in the development of tolerance [29, 124]. Conversely, human transplantation studies have demonstrated that maternal microchimerism may result in sensitization and subsequent rejection as seen in the higher rates of acute rejection in kidney and stem cell NIMA mismatched grafts [125, 126]. Lastly, fetal microchimerism has been identified in maternal cancers including breast, papillary thyroid, and lung cancer. However, the function of fetal cells in these cancers are not clearly defined and may include tumorigenesis, immune surveillance and tissue repair [18].

## 4. Lessons Learned from Fetomaternal Tolerance for Transplantation

Understanding the mechanisms of fetomaternal tolerance may provide new insights into developing methods of preventing transplant allograft rejection. For the translation of fetomaternal tolerance to the field of transplantation, we can parallel the hypotheses of Medawar. Anatomical separation between mother and fetus by the placenta. Anatomic separation of allograft is under study in the field of islet transplantation using incapsulated islets to physically block host immunity from interacting with transplanted islets [127]. Hypothetically, islets could also be placed in areas of “immune privilege”, such as in the cerebral spinal fluid, testis, or uterus. Such methods may work for other smaller allograft, such as islets or parathyroid tissue, but may be difficult to achieve for larger, solid organ allografts. Immaturity of fetal antigens relates to impairing the ability of the graft to present antigen and to elicit an immune response. To some degree this has been achieved by decreasing effective allograft antigen presented by blocking costimulation signals with CTLA4-Ig [128]. Graft depletion of donor leukocytes is another method that will decrease donor antigen presentation and is currently in clinical use for small bowel transplantation where transplant donors are treated with thymoglobulin prior to organ procurement [129, 130]. New research is targeting graft MHC with small interfering RNAs (siRNAs) to down-regulate donor MHC [131]. Of course, a caveat to MHC depletion is that it can stimulate a natural killer cell mediated immune response against the graft. Immunological inertness of the maternal immune system during pregnancy. This is our current method of immunosuppression, inhibiting recipient immune responses through the use of immunosuppressive medications (e.g. prednisone, calcineurin inhibitors, mTOR inhibitors, cell cycle inhibitors and recipient lymphocyte depletion). Methods are being developed that may allow specific inhibition of immune responses against donor tissue, rather than global immunosuppression. For example, methods of central tolerance are being tested that use donor bone marrow transplantation to produce donor-recipient chimerism [132], and also donor thymus co-transplantation [133]. In addition, methods for obtaining peripheral tolerance through donor leukocyte infusions [134] and Treg infusions [135, 136] have been studied with some success. Recently, mesenchymal cell transplantation has emerged as a promising strategy for tolerance induction [137].

Fetomaternal interactions at the placenta and cell trafficking that occurs through the placenta have significant clinical implications that extend outside of transplantation. As indicated above, fetal and maternal microchimerism may promote either tolerance or immunogenicity in autoimmune disease, transplantation, and cancer. The balance between tolerance and immunogenicity represents a mechanism, which can be further harnessed to promote clinical advances in autoimmunity, transplantation, and oncology.

## 5. Summary

Despite significant advances in operative technique, organ storage, and immunosuppressive medications, we continue to suffer persistent graft losses due to acute and chronic immune-medicated rejection. Developing methods that produce donor-specific immune tolerance or create effective immune barriers to the alloimmune response would provide alternate modalities for improving the longevity of donor organs. The fetomaternal barrier has evolved to achieve immune tolerance to foreign tissue using multiple layers of protection. New approached capable of leveraging the mechanisms of fetomaternal tolerance may lead to improved methods of immunosuppression and could ultimately produce a tolerant interface between the recipient immune system and donor organs.

## Figures and Tables

**Table 1:** Summary of HLA expression, immune cells, and cytokines.

**HLA**	
Class I (fetus)	HLA-C, HLA-E, HLA-F, HLA-G
Class II (fetus)	None
**Cells**	
Regulatory T cells	High
Effector T cells	Low^1^
CD8+ cytotoxic T cells	Low^1^
NK cells	High
Macrophages	High
**Cytokines**	
IL-10	High
TGF-3	High
VEGF	High
IDO	High
MMP9	High
MIP	High
GM-CSF	High
Fas-L	High
TRAIL	High

1 Increase in third trimester [36, 37]. MMP9: matrix metallopeptidase 9.

**Table 2:** Summary of maternal hormone, immune cell, and cytokine changes during pregnancy.

	First Trimester	Second Trimester	Third Trimester
**Hormones**			
Estrogens	Low	Increasing	High
Progesterone	Low	Increasing	High
HCG	High	Decreasing	Low
**Immune Cell Populations vs Postpartum Profile**			
CD4+ and CD8+ T cells	Low	Low	Low
Th Cells	Low	Low	Low
B Cells	Normal	Normal	Low
NK cells	High	Decreasing	Low
pDCs and mDCs	High	High	High
**Cytokine Levels vs Postpartum Levels**			
TNF-α	High	High	High
G-CSF	High	High	High
IL-15	High	High	High
IFN-γ	Normal	Low	Low
VEGF	Low	Low	Low
MCP-1	Low	Low	Low
Eotaxin	Low	Low	Low
IL-2	High	Normal	Normal
